# A review of the preclinical and clinical studies on the role of the gut microbiome in aging and neurodegenerative diseases and its modulation

**DOI:** 10.3389/fncel.2022.1007166

**Published:** 2022-11-03

**Authors:** Haslin Madihah Hashim, Suzana Makpol

**Affiliations:** Department of Biochemistry, Faculty of Medicine, Universiti Kebangsaan Malaysia Medical Centre, Kuala Lumpur, Malaysia

**Keywords:** aging, gut microbiome, brain-gut-microbiota axis, neurodegenerative diseases, interventions

## Abstract

As the world population ages, the burden of age-related health problems grows, creating a greater demand for new novel interventions for healthy aging. Advancing aging is related to a loss of beneficial mutualistic microbes in the gut microbiota caused by extrinsic and intrinsic factors such as diet, sedentary lifestyle, sleep deprivation, circadian rhythms, and oxidative stress, which emerge as essential elements in controlling and prolonging life expectancy of healthy aging. This condition is known as gut dysbiosis, and it affects normal brain function via the brain-gut microbiota (BGM) axis, which is a bidirectional link between the gastrointestinal tract (GIT) and the central nervous system (CNS) that leads to the emergence of brain disorders such as Alzheimer’s disease (AD), Parkinson’s disease (PD), amyotrophic lateral sclerosis (ALS), and frontotemporal dementia (FTD). Here, we reviewed the role of the gut microbiome in aging and neurodegenerative diseases, as well as provided a comprehensive review of recent findings from preclinical and clinical studies to present an up-to-date overview of recent advances in developing strategies to modulate the intestinal microbiome by probiotic administration, dietary intervention, fecal microbiota transplantation (FMT), and physical activity to address the aging process and prevent neurodegenerative diseases. The findings of this review will provide researchers in the fields of aging and the gut microbiome design innovative studies that leverage results from preclinical and clinical studies to better understand the nuances of aging, gut microbiome, and neurodegenerative diseases.

## Introduction

**FIGURE 1:**
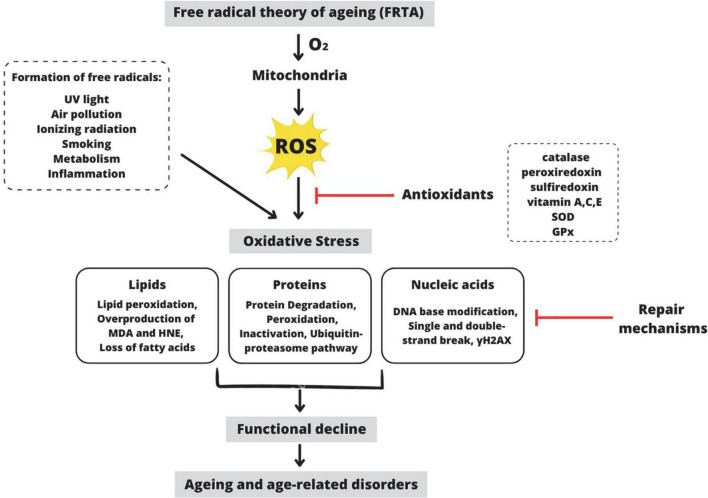
Schematic representation of the premise behind the Free Radical Theory of Aging (FRTA). Oxygen toxicity is the basis of FRTA. By-radical molecular oxygen can generate partially depleted molecules and ROS (Balaban et al., 2005). ROS are primarily produced during oxidative phosphorylation in the mitochondria, but they are also produced by other exogenous and endogenous factors such as ultraviolet light, air pollution, etc. Catalase, vitamin A, SOD, and other antioxidants can detoxify ROS within the cell (Davies, 2000). When these antioxidants are depleted, ROS accumulates, disrupting the cell’s normal redox state and resulting in oxidative stress. ROS-induced oxidative stress causes overproduction of MDA and HNE, which act as a second messenger of oxidative stress and a major bioactive marker of lipid peroxidation (Barrera, 2012). Additionally, oxidative stress causes protein degradation or proteolysis via the ubiquitin-proteosome pathway (Cooper, 2000). Moreover, oxidative stress also causes DNA base modification via γH2AX, a molecular marker of DNA damage and repair (Mah et al., 2010). Despite mechanisms for repairing oxidatively damaged biomolecules, several damages remain. According to FRTA, oxidative stress causes aging, physiological decline, and age-related disorders (Selman et al., 2012). ROS, reactive oxygen species; SOD, superoxide dismutase; GPx, glutathione peroxidase; MDA, malondialdehyde; HNE, 4-hydroxynonenal; γH2AX, phosphorylated histone H2AX. This figure was created with Canva.com.

Aging is unavoidable in the human life cycle, characterized by progressive physiological decline, leading to increased frailty, disease, and decreased longevity (Hou et al., 2019). Gerontology is the study of the aging process, which involves a complex interaction of behavior, chemistry, genetics, and physiology. There are now dozens of aging theories explaining why aging is inevitable. The free radical theory of aging (FRTA), which Denham Harman first proposed in the 1950s, has become one of the most prominent theories to explain aging (Figure 1; Harman, 1956). This theory proposes that the rate of oxidative damage to mitochondrial DNA determines life span primarily. For many decades, FRTA has established a theoretical basis for extensive studies and received abundant support from scientific research, resulting in significant advancements in our knowledge of aging. Past studies revealed a correlation between reducing oxidative stress and extending the lifespan in various model organisms, including nematodes (Han et al., 2017), African turquoise killifish (Smith et al., 2017), naked mole-rat (Debebe et al., 2017), fruit flies (Shenghua et al., 2020), and mice (Wang et al., 2020). Extensive scientific evidence supports the FRTA, which is manifested in the levels of oxidative stress to the damage in specific molecules including lipids, proteins, and mitochondrial DNA (Hajam et al., 2022). As a result, with regard to free radicals in aging, it has advanced to the point of becoming one of the more reasonable theories of the aging process.

Free radicals such as reactive oxygen species (ROS) are essential electron donors in normal metabolism. ROS is produced by both exogenous and endogenous sources, such as mitochondrial oxidative metabolism and ionizing radiation. ROS promotes inflammation, accelerates aging, and increases the risk of neurodegenerative diseases (Thanan et al., 2014). Under a normal state, free radicals are needed for primary biological responses, including gene transcription, leukocyte adherence, platelet accumulation, signal transduction, and smooth muscle relaxation. Cells produce excess free radicals when exposed to ROS, which can be neutralized by cell-induced antioxidants, for instance, superoxide dismutase (SOD). When the production of free radicals exceeds detoxification capacity, oxidative damage occurs, which can amplify DNA mutations and lead to mitochondrial dysfunction or apoptosis (Buehler, 2012).

Aging has become a significant predictor of neurodegeneration, and aggregation of oxidative damage to mitochondrial DNA may be related to neurodegenerative diseases (Hou et al., 2019). As part of the ATP production process, mitochondria are involved in several intercellular signaling pathways, including calcium signaling, biosynthesis of lipids, and programmed cell death (Keogh and Chinnery, 2015). Furthermore, tissues made predominantly of postmitotic cells (e.g., the brain) are vulnerable to the development of aging because they are more susceptible to DNA damage than accumulating cells (Hou et al., 2019). Interestingly, organ function decline with age, resulting in a gradual decline in physical and mental function, such as blurred vision, hearing loss, and muscle atrophy (Inouye et al., 2007).

Simultaneously, aging is associated with the inability to accelerate a robust immune response, a condition known as immunosenescence, and with age-related inflammation, a condition known as inflammaging (Conway and Duggal, 2021). Mild inflammation (inflammaging) is associated with a chronic inflammatory disease affected by changes in intestinal microbiome composition, which is defined by its instability and diversity (Dinan and Cryan, 2017). This causes the breakdown of the gut barrier, increased pro-inflammatory cytokines levels and bacterial byproducts in the bloodstream, damage to the blood-brain barrier (BBB) and neuroinflammation (Köhler et al., 2016) that leads to cognitive decline, frailty, metabolic disease, and mortality (Ferrucci and Fabbri, 2018).

A substantial amount of research has been conducted on the role and abundance of the intestinal microbiome as well as the implications for maintaining a healthy state. Gut microbiota (GM) is an ecosystem metabolic of a million different microorganisms living in the gastrointestinal tract (GIT) and forming a symbiotic connection with the host (Cornejo-Pareja et al., 2019). Because GM helps to maintain physiological homeostasis, alterations in microbiome abundance taxa cause intestinal dysbiosis related to numerous pathological conditions, including neurodegenerative diseases (Westfall et al., 2017; Juárez-Fernández et al., 2021). Thus, microbiota-based therapies emerge as a potential therapeutic target, including prebiotic or probiotic administration, nutrition and physical activity to reshape the GM (Juárez-Fernández et al., 2021). In the present review, we aim to address the role of gut microbiome in aging and neurodegenerative diseases, with a particular focus on targeting the gut microbiome as an intervention to slow the aging progression and prevent neurodegenerative diseases.

## The role of gut microbiome in aging

Gut microbiota is a diverse group of microorganisms present in the GIT, and its genes are known as the microbiome (Salazar et al., 2020). Each individual has a unique microbiota composition that is very diverse and complex in nature (Balan et al., 2021), which is influenced by biological factors such as genetics and lifestyle factors including dietary (Salazar et al., 2019), exercise (Huang et al., 2019), sleep deprivation (Smith et al., 2019), drugs (Sun et al., 2019c), and mental health (Barandouzi et al., 2020). The Human Microbiome Project (HMP) and Metagenomics of the Human Intestinal Tract (MetaHIT) have currently revealed an extensive view of the intestinal microbiota in healthy people, which is made up of permanent and transient microbial species and subspecies from over 17 different phyla belonging to Firmicutes [*Clostridium*, *Eubacterium*, *Faecalibacterium*, *Lactobacillus, Roseburia*, and *Ruminococcus*], Bacteroidetes [*Bacteroides* and *Prevotella*], Proteobacteria [*Escherichia, Helicobacter, Salmonella*, and *Shigella*], Actinobacteria [*Bifidobacterium*], Fusobacteria, Spirochaetes, Verrucomicrobia, Lentispherae, and other phyla (Lloyd-Price et al., 2016, 2017; Parks et al., 2017).

Several research revealed that the composition and stability of the intestinal microbiome change significantly with aging (Salazar et al., 2017; Nagpal et al., 2018). Firmicutes are enriched during childhood and adolescence, while with increasing age, Bacteroidetes become the dominant phylum (O’Toole and Jeffery, 2018). Several studies have reported that the prevalence of major commensal organisms, including *Bacteroides*, *Bifidobacterium*, and *Lactobacilli*, decreases in old people (Santoro et al., 2018; Salazar et al., 2019). Rahayu et al. (2019) studied the GM of 80 healthy Indonesians, divided into two groups: young (25–45 years) and elderly (70 years). The findings revealed that the gut microbiome composition was higher in the young than in the elderly, with *Atopobium*, *Bifidobacterium*, *Bacteroides, Clostridium*, and *Prevotella* being the most common bacterial groups, while an increase in Enterobacteriaceae and *Escherichia coli* was found in the elderly. Moreover, Kim B. S. et al. (2019) studied 56 South Korean subjects divided into centenarians, elderly, and adults, and discovered that centenarians had a higher abundance of Firmicutes than the elderly and adults, while Bacteroidetes were lower. Furthermore, a shift in the centenarian’s microbiota, with lower abundances of *Faecalibacterium* and *Prevotella* and higher proportions of *Akkermansia*, *Clostridium*, *Collinsella*, and *Escherichia*. Similarly, Wang N. et al. (2019) showed that the GM of East China centenarians is composed of several microbes that may influence their lifespan, including *Bacteroides fragilis*, *Clostridium perfringens*, *Parabacteroides merdae*, and *Ruminococcus gnavus*. Indeed, the gut microbiome is dynamic across a lifetime.

In general, these alterations are known as “gut dysbiosis,” which is distinguished by a decrease in a plethora of beneficial microorganisms, including bacteria that generate short-chain fatty acids (SCFAs), e.g., *Clostridium*, *Bifidobacterium*, *Lactobacillus*, and *Roseburia* (Kim and Jazwinski, 2018). Gut microbes produce lots of SCFAs, mainly butyrate, acetate, and propionate, which are immunomodulatory bacterial metabolites. SCFAs act as messengers between the gut microbiome and the immune response by transmitting signals through free fatty acid receptors (FFARs), which are members of the G protein-coupled receptors (GPCRs) (Ratajczak et al., 2019). SCFAs bind to GPCRs, including GPR109A, GPR41, and GPR43, expressed on the membrane of epithelial and immune cells. SCFA is transported within host cells and inhibits histone deacetylase (HDAC) activity. SCFAs improved gut barrier function and immune sensitivity via several mechanisms, including: (i) increased mucus production by gut goblet cells, (ii) nuclear factor-κB (NF-κB) repression, (iii) activation of inflammasomes, (iv) production of interleukin-18 (IL-18), (v) increased secretory IgA (sIgA) by B cells, (vi) decreased T cell-activating molecules expression on dendritic cells, (vii) increased FOXP3 expression, (viii) increase anti-inflammatory cytokines such as interleukin 10 (IL-10) and transforming growth factor-β (TGFβ) (Rooks and Garrett, 2016). Furthermore, these SCFA properties influence their immunomodulatory activity, such as maintaining the stability of pro-and anti-inflammatory immune biomarkers. They also have antioxidant, anticancer, and anti-inflammatory properties, which help to maintain immune homeostasis in the CNS (Corrêa-Oliveira et al., 2016; Riaz Rajoka et al., 2018; Ratajczak et al., 2019).

Lipopolysaccharide (LPS) is an inflammatory toxin produced by certain microbes, such as *Bacteroides* and *Prevotella*. LPS stimulates the TLR4 receptor by interacting with CD14 and MD-2 proteins, triggering an inflammatory response (Zhao et al., 2015). According to other studies, LPS produced by *Bacteroides fragilis* activates the pro-inflammatory transcription factor NFκB, which is responsible for the progression of AD in microglial cells. NFκB stimulates the pro-inflammatory micro RNA (miRNAs) transcription, including miRNA-155, miRNA-146a, miRNA-125b, miRNA-34a, and miRNA-9, which activates neuroinflammatory mediators and prevents phagocytosis (Zhao and Lukiw, 2018). For instance, it was recognized that miRNA-34a reduces the expression of TREM2, which is the activating receptor expressed on microglia cells, impairing microglia phagocytosis and enhancing amyloid β-42 (Aβ42) aggregation (Bhattacharjee et al., 2016). Recently, a clinical trial in Alzheimer’s disease (AD) patients revealed that the present bacterial LPS in the brain causes LPS levels in the neocortex and hippocampus to increase by two and three-folds, respectively (Zhao et al., 2017). However, excess LPS from the gut entering the bloodstream may cause inflammation by breakdown of the gut barrier (“leaky gut”), allowing LPS and pathogenic microbes to enter the bloodstream. Thus, elevated LPS levels and blood inflammation have been linked to a variety of brain disorders, such as dementia, major depression, and schizophrenia (Kelly et al., 2015).

Bacterial amyloids such as curli secreted by pathogenic bacteria such as *Escherichia coli*, S*almonella enterica*, and *Bacillus subtilis* (Hufnagel et al., 2013; Schwartz and Boles, 2013) may prime the immune system, increasing immune response to endogenous neuronal amyloid production in the brain (Friedland and Chapman, 2017). Amyloid peptide is in charged of a number of physiological mechanisms, such as bacterial cell binding and biofilm formation, and resistance to immune factors (Evans et al., 2018). Curli peptide has a β-folded sheet secondary structure and stains with Congo red and thioflavin, which are dyes used to stain the amyloid deposits in the brain. It has been discovered that the precursor of amyloid gA has a structure similar to Aβ42 and is able to be identified by the TLR2 receptor. Therefore, binding of TLR2 with curli peptide activates macrophages and produces the pro-inflammatory cytokines IL-1β and IL-6 (Rapsinski et al., 2015). Similarly, microbial amyloid has been found to stimulate *T*-lymphocytes and trigger the production of pro-inflammatory interleukins IL-17A and IL-22 (Nishimori et al., 2012). Both cytokines have the ability to cross the blood brain barrier (BBB) and trigger ROS production as well as activate TLR2/1 and NFκB signaling pathways in microglia and astrocytes, leading to neuroinflammation and neurodegeneration (Perriard et al., 2015; Sun et al., 2015; Zhan et al., 2018). According to Chen et al. (2016), oral infectious disease of aged rats with curli-producing *E. coli* resulted in increased α-syn deposits in brain tissue microgliosis and astrogliosis as well as enhanced expression of TLR2, IL6, and TNF.

Several studies have revealed that the gut microbiome modulates Th17 cells and Treg cells, implying that the microbiome composition has a significant impact on the immune responses against pathogenic microbes and inflammatory responses (Park et al., 2015). The involvement of segmented filamentous bacteria (SFB) such as Actinobacteria, Bacteroidetes, Firmicutes, and Proteobacteria phyla in the gut has been linked to the induction of Th17-mediated autoimmune disorders (Yi et al., 2010; Lee et al., 2011). SFB protects against pathogenic microbes such as *Citrobacter rodentium* by triggering production of IL-22 by Th17, which inhibits its growth (Ivanov et al., 2009). Similarly, SFB protects non-obese diabetes (NOD) mice from developing type-1 diabetes (T1D) in an IL-17-dependent manner (Kriegel et al., 2011). Furthermore, SCFAs also promote the differentiation of T lymphocytes into Th1 and Th17, which play a role in pathogen defense and mediate the inflammatory response (Ratajczak et al., 2019). Acetate and propionate have been shown to induce naive T lymphocytes into T helper 17 (Th17) cells and to stimulate the development of T helper type 1 (Th1) cells via interleukin 12 (IL-12) (Park et al., 2015; Ratajczak et al., 2019). Xu et al. (2018) revealed that *Helicobacter hepaticus* is also involved in inflammatory bowel diseases by inducing Th17 proinflammatory lymphocytes. According to this study, the inactivation of the transcription factor c-MAF in the Treg lymphocytes disrupted their differentiation and function, reducing IL-10. Numerous studies have proven the important role of gut microbiome in aging and neurodegenerative diseases (Table 1). Over the last decade, many studies have been carried out on the effects of gut microbiome on the CNS, and the concept of a “brain-gut-microbiota axis” has been introduced (Kowalski and Mulak, 2019).

**TABLE 1:** Role of the gut microbiome on aging and neurodegenerative diseases.

No.	References	The role of the gut microbiome on aging and neurodegenerative diseases	Abundance of bacteria
1	Badal et al., 2020	Promotes gut homeostasis and healthy aging by lowering adiposity, inflammation, and the risk of developing metabolic and cognitive dysfunction.	↑ Verrucomicrobia ↑ *Akkermansia* ↑ *Christensenellaceae*
2	Sorbara and Pamer, 2019	Disrupt the intestinal barrier integrity and causes chronic inflammation, further aggravating microbial dysbiosis and increasing susceptibility to gastrointestinal infections.	↑ *Clostridium difficile* ↑ *Helicobacter pylori*
3	Dominy et al., 2019	It could cause accumulation of amyloid-beta plaques and neurofibrillary tangles.	↑ *Porphyromonas gingivalis*
4	Junges et al., 2018	Involved in the synthesis of aminobutyric acid (Y-Aminobutyric acid, GABA).	↑ *Bifidobacterium* ↑ *Lactobacillus*
5	Strandwitz, 2018	It causes brain dysfunction, which is characterized by synaptogenesis disorders, depression, and cognitive impairment.	↓ *Bifidobacterium* ↓ *Lactobacillus*
6	Xu et al., 2018	It involved in inflammatory bowel diseases by inducing Th17 proinflammatory lymphocytes.	↑ *Helicobacter hepaticus*
7	Rizzatti et al., 2017	Increased gut inflammation and dysbiosis.	↑ Proteobacteria
8	Louis and Flint, 2017	It is crucial in the production of the SCFA butyrate.	↑ *Faecalibacterium*
9	Chudnovskiy et al., 2016	It defends against enteric bacterial infection by activating epithelial inflammasome signaling and promoting DC-driven Th1 and Th17 immunity.	↑ *Trichomonas musculis*
10	Seo et al., 2015	It stimulates monocytes to release NLRP3-dependent IL-1β, which causes intestinal inflammation.	↑ *Proteus mirabilis*
11	Divyashri et al., 2015	Reduce TNF-α production, oxidative stress markers, and induced antioxidant enzymes in the brain.	↑ *Enterococcus faecium* ↑ *Lactobacillus rhamnosus*
12	Zhao et al., 2015	Produces LPS and activates the pro-inflammatory transcription factor NFκB.	↑ *Bacteroides fragilis*
13	Liang et al., 2015	Increased production of IL-10, restored levels of norepinephrine and serotonin, and enhanced BDNF expression in the hippocampus.	↑ *Lactobacillus helveticus NS8*
14	Bischoff et al., 2014	Enhance the intestinal barrier by increasing the expression of proteins that forming tight junctions.	↑ *Lactobacillus plantarum* ↑ *Escherichia coli Nissle* ↑ *Bifidobacterium infantis*
15	Hufnagel et al., 2013	It is capable of secreting large quantities of the bacterial amyloid peptide *curli*.	↑ *Escherichia coli* ↑ *Baccilus subtilis* ↑ *Salmonella tyrhimurium* ↑ *Salmonella enterica*

Changes (↑: increase; ↓: decrease) in the relative abundance of selected microbial taxa.

## Brain-gut-microbiome axis and neurotransmitters

There is now a great deal of understanding about the connection between the intestinal microbiome and brain functions (Askarova et al., 2020). The brain-gut-microbiome (BGM) axis is a communication network that connects the gut and brain (Cryan et al., 2019) via three basic mechanisms mediating gut-brain communication, including direct neural communication, endocrine signaling mediators, and the immune system, which is dedicated to the progression of neurodegeneration and neuroinflammation (Bauer et al., 2016). The gut and the brain are linked by millions of nerve cells, specifically the vagus nerve, which sends signals in both directions (Bonaz et al., 2018). The gut microbiome transmits information from ingested components passing through the GIT, such as vitamins, minerals, carbohydrates, and fats, to the CNS via pathways to induce a systemic response associated with the reflected dietary and energy conditions (Noble et al., 2017). When there is gut dysbiosis, messages sent to the brain send out unhealthy signals that indicate mild inflammation, increased oxidative damage, an imbalance in energy homeostasis, and an overall improvement in cellular neurodegeneration (Noble et al., 2017). The gut microbiome impacts brain functions, impaired BBB, altered synaptic plasticity, microglial activity, neurogenesis, neurotransmitter production, and behavioral effects (Luczynski et al., 2016; Chu et al., 2019). According to recent research, the intestinal microbiome significantly impacts the pathogenesis of depression symptoms in rats via the BGM axis (Zhang et al., 2022). Furthermore, changes in the BGM axis may also influence the progression of neurodegenerative diseases (Kowalski and Mulak, 2019).

The gut and the brain are also linked by chemicals known as neurotransmitters, which help in monitoring and integrating gut functions with the cognitive and emotional functions of the brain (Scriven et al., 2018). Interestingly, the gut microbiome can produce a variety of mammalian neurotransmitters such as dopamine (*Bacillus*, *Escherichia*, *Lactobacillus*, *Lactococcus*, and *Streptococcus*), serotonin (*Escherichia*, *Enterococcus*, *Lactobacillus*, and *Streptococcus*), acetylcholine (*Lactobacillus* and *Bacillus*), noradrenaline (*Bacillus* spp.), norepinephrine (*Bacillus*), histamine (*Lactobacillus*, *Lactococcus*, *Streptococcus*, and *Enterococcus*), and γ-aminobutyric acid (GABA; *Bifidobacterium* and *Lactobacillus*), all of which affect the host’s well-being and maintain homeostasis (Alkasir et al., 2016; Strandwitz, 2018). GABA is an amino acid that acts as an inhibitory neurotransmitter, and it was found that the aminobutyric acid levels in the gut coincide with those in the CNS (Strandwitz, 2018). For instance, GABA helps regulate feelings of fear and anxiety (Mazzoli and Pessione, 2016), and a study in laboratory mice revealed that certain probiotics could enhance GABA production, thereby reducing anxiety and depression-like behavior (Janik et al., 2016).

Brain-derived neurotrophic factor (BDNF) acts as a neurotransmitter modulator that is involved in synaptic plasticity, which is extremely important for all forms of learning and memory. There is evidence that Alzheimer’s disease patients have lower BDNF levels in their brains and serum (Michalski et al., 2015). *Lactobacillus helveticus NS8* also lowered hormone levels such as corticosterone and adrenocorticotropic in plasma, enhanced production of IL-10, restored levels of norepinephrine and serotonin, and enhanced BDNF expression in the hippocampus (Liang et al., 2015). Similarly, the *N*-methyl-D-aspartate (NMDA) receptor is triggered by a glutamate excitatory neurotransmitter and is also involved in synaptic plasticity. Wang et al. (2015) demonstrated that the use of ampicillin for 1 month in rats resulted in gut dysbiosis, which reduced NMDA receptor expression, increased aggressive behaviors, and impaired spatial cognition, whereas introducing the *Lactobacillus fermentum NS9* strain into the gut microbiome normalized these conditions. As a result, imbalances in these neurotransmitters can induce both psychological and neurological disorders.

## Mechanisms of the effects of the gut microbiome on the pathogenesis of neurodegenerative diseases

**FIGURE 2:**
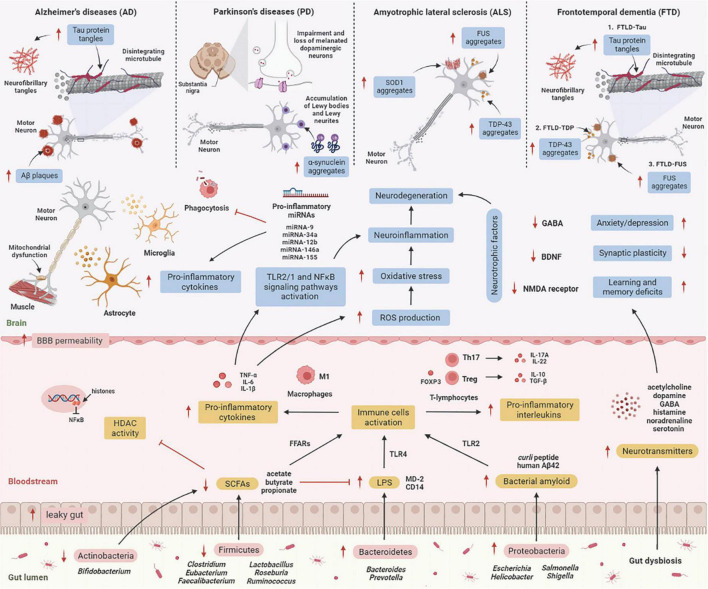
The mechanism underlying the effect of the gut microbiome on the etiology of neurodegenerative diseases. GIT is composed of a diverse group of microbes, and its composition changes significantly with age. These alterations are termed “gut dysbiosis,” which leads to increased leaky gut, causing translocation of bacteria (a process known as atopobiosis) into the bloodstream (König et al., 2016). Reduced numbers of beneficial microbes that produce SCFAs such as Firmicutes and Actinobacteria is unable to inhibit HDAC activity and LPS-induced inflammation. On the other hand, gut microbes such as Bacteroidetes are able to excrete an abundance of LPS, which stimulates the TLR4 receptor by interacting with CD14 and MD-2 proteins, triggering an inflammatory response (Zhao et al., 2015). Furthermore, Proteobacteria produce an abundance of bacterial amyloids such as *curli* peptide, and binding of *curli* peptide to TLR2 activates macrophages, which secrete pro-inflammatory cytokines such as TNF-α, IL-6, and IL-1β (Rapsinski et al., 2015), and activation of T-lymphocytes induces the production of pro-inflammatory interleukins such as IL-17A and IL-22 by Th17 cells (Nishimori et al., 2012). These cytokines are able to penetrate the BBB, increase production of ROS, and promote oxidative stress, leading to neuroinflammation and neurodegeneration (Zhan et al., 2018). These cytokines also activate TLR2/1 and NFκB signaling pathways in microglia and astrocytes, which stimulates the transcription of pro-inflammatory miRNAs, activates neuroinflammatory mediators, and inhibits phagocytosis in microglial cells (Zhao and Lukiw, 2018), leading to the progression of neurodegenerative diseases. [AD: increased Aβ plaques and P-tau tangles; PD: increases α-synuclein aggregates in Lewy bodies and Lewy neurites, and impairment and loss of melanated dopaminergic neurons in the substantia nigra; ALS: increases SOD1, FUS, and TDP-43 aggregates; TDP: increases P-tau tangles, TDP-43 and FUS aggregates]. Additionally, several microbes may signal through their metabolites to promote the synthesis and release of neurotransmitters, which are involved in the transport of chemical signals from nerve cells to the target cell, such as muscle or gland. Gut dysbiosis may also decrease synthesis and secretion of neurotrophic factors such as GABA, BDNF, and NMDA receptors, leading to neurodegeneration (Askarova et al., 2020). Aβ, amyloid-beta; BBB, blood-brain barrier; BDNF, brain-derived neurotrophic factor; CD14, cluster of differentiation 14; FFARs, free fatty acids receptors; FOXP3, forkhead box P3; FUS, fused in sarcoma; GABA, gamma-aminobutyric acid; GIT, gastrointestinal tract; HDAC, histone deacetylase; IL, interleukin; LPS, lipopolysaccharide; NF-κB, nuclear factor-κB; MD-2, myeloid differentiation factor-2; NMDA receptor, *N*-methyl-D-aspartate receptor; SCFAs, short-chain fatty acids; SOD1, superoxide dismutase 1 gene; TDP-43, TAR DNA-binding protein 43; TGF-β, transforming growth factor-β; Th, T helper; TLR, tall-like receptor; TNF-α, tumor necrosis factor alpha; Treg, regulatory T cells. This figure was created with BioRender.com.

Numerous molecular research discovered a relationship between gut microbes and neurological disorders known as neurodegenerative diseases, in which patients with elevated intestinal inflammation having lower microbiome diversity than healthy cohorts with relatively intact abundance (Rowin et al., 2017). Each neurodegenerative disease has a distinct clinical aspect and pathology. Molecular research has revealed that the brain tissue of the elderly consists of abnormal deposits of proteins such as amyloid-β (Aβ), hyperphosphorylated tau (p-tau), or α-synuclein (α-syn) (Elobeid et al., 2016). Despite this, it is unclear how the gut microbiome influences these deposits in the brain, resulting in neurodegenerative diseases. Here, we describe the mechanism underlying the effects of gut microbiome on the etiology of neurodegenerative diseases (Figure 2).

Alzheimer’s disease (AD) is distinguished by a gradual deterioration in neuronal function (Jiang et al., 2017). The primary neuropathological features of AD are the aggregation of the amyloid-β (Aβ) plaques outside of neurons and neurofibrillary tangles (NFT) composed of hyperphosphorylated tau (p-tau) protein inside neurons in the brain (Kowalski and Mulak, 2019). These deposits trigger neuroinflammation, which eventually leads to synaptic deterioration and neuronal death (Köhler et al., 2016). Mutations in three genes, including presenilin gene 1 (PS1) on chromosome 14, presenilin 2 gene (PS2) on chromosome 1, and amyloid precursor protein gene (APP) on chromosome 21, are responsible for transmitting AD via autosomal-dominant inheritance. Early-onset AD (EOAD) is usually diagnosed before 65 years old, whereas the sporadic type of disease, known as late-onset AD (LOAD), appears in people over the age of 65 (Panegyres and Chen, 2013, 2014). A diverse gut microbiome promotes functional amyloids in the AD brain via bacterial amyloids such as *Escherichia coli*, *Streptomyces*, *Bacillus*, *Pseudomonas*, and *Staphylococcus*, known as “curli fibers,” which are made up of the major curli subunit protein CsgA, which aids bacterial cells bind together to form biofilms and host defense protection from immune factors (Hill and Lukiw, 2015). According to a clinical study, the gut microbiome proportion of AD patients significantly differs compared to healthy people, with Firmicutes and *Bifidobacterium* being reduced and Bacteroidetes being increased (Brunt et al., 2019).

Parkinson’s disease (PD) is defined by dysfunctional motor neurons and neuropsychiatric signs. The pathogenesis features of PD involve neuronal degeneration in the substantia nigra (SN) due to proteostasis of α-synuclein, oxidative damage, mitochondrial dysfunction, impaired axonal transport, calcium homeostasis, and neuroinflammation (Poewe et al., 2017). Concurrently, these features result in striatal dopamine deficit and intracellular aggregates consisting of α-synuclein deposits, manifesting as locomotor signs, such as neuromuscular dysfunctions affecting movement speed, muscle stiffness and resting tremor (Hou et al., 2019). The earliest PD-linked genetic discovery was made in 1997, with the discovery of a missense mutation in synuclein alpha (α-syn) (Polymeropoulos et al., 1997). Up to this point, function loss variants in about 20 genes have now been linked with PD, including PINK1, PRKN, PARK7, LRRK2, PLOG, and GBA (Blauwendraat et al., 2022). Chen et al. (2016) proposed that amyloid proteins in the microbiome of the intestine are responsible for the onset and progression of neurological conditions. According to this study, curli secreted by *E. coli* caused enhanced α-syn deposits in the brain as well as increased astrogliosis and microgliosis in rats. Kim S. et al. (2019) support the Braak hypothesis in the pathogenesis of undiagnosed PD by injecting α-syn fibrils into the gut, which convert intrinsic α-syn into a pathologic organism that spreads to the brain. This results in PD-like symptoms, vagotomy and, α-syn deficit, which inhibits the neuropathology and neurobehavioral problems caused by pathological α-syn transmission. Clinical research by Aho et al. (2019) revealed that the variations in GM of 64 PD patients and 64 control subjects persisted after 2 years, with *Prevotella* and *Roseburia* being reduced and *Bifidobacterium* being increased.

Amyotrophic lateral sclerosis (ALS) is related to motor neuron damage in the spinal cord due to muscle frailty, atrophy, and spasticity (Hardiman et al., 2017). Several genetic variants in non-neuronal cells have been linked to the pathogenesis of ALS, including the 43-kDa TAR DNA-binding protein (TDP-43) and superoxide dismutase 1 (SOD1), and C9orf72 and the expression of these genes are linked to immunological neuroinflammation in ALS (Beers and Appel, 2019). Patients with familial ALS typically develop the disease younger than those with sporadic ALS (Aktekin and Uysal, 2020). The current study discovered a new relationship between the microbiome, hSOD1^G93A^ accumulation, and gut mobility in SOD1^G93A^ mice, with longitudinal studies of microbiome data revealing a shift in gut microbiome composition related to autoimmunity (*Clostridium* sp. and *Lachnospiraceae bacterium*), inflammation (*Enterohabdus muris*), and metabolism (*Desulfovibrio fairfieldensis*) (Zhang et al., 2021). Clinical research by Nicholson et al. (2021) found that the proportional abundance of butyrate-producing microbes, including *Eubacterium rectale* and *Roseburia intestinalis*, was significantly decreased in ALS patients, indicating that these levels of butyrate-producing bacteria are significant for intestinal barrier and inflammation control. Other clinical studies revealed significant changes in the microbiome composition of ALS patients, with Bacteroidetes being up-regulated and Firmicutes being down-regulated at the phylum level when compared to healthy controls (Zeng et al., 2020).

Frontotemporal dementia (FTD) is a type of dementia with neuropathological features involving chronic atrophy in the frontal and neocortex as well as the accumulation of microtubule-associated protein tau and two RNA-binding proteins, 43-kDa TAR DNA-binding protein (TDP-43) and fused in sarcoma (FUS) (Hoffman et al., 2019). FTD is associated with various genetic etiologies, the most prevalent being accounted for by autosomal dominant mutations in the chromosome 9 open reading frame 72 (C9orf72), progranulin (GRN), and microtubule-associated protein tau (MAPT) genes (Fenoglio et al., 2018). It was previously reported that lowered C9orf72 activity resulted in inflammatory responses distinguished by hypercytokinemia, neutrocytosis, spurious thrombocytopenia, systemic sclerosis, splenomegaly, and neuroinflammation (Atanasio et al., 2016; Burberry et al., 2016; Jiang et al., 2016; O’Rourke et al., 2016). Following these findings, it was later discovered that FTD patients with C9orf72 mutations had a substantially increased risk of being diagnosed with an autoimmune disorder prior to their brain condition (Miller et al., 2016; Fredi et al., 2019). Burberry et al. (2020) found that reducing the abundance of immune-stimulating bacteria such as *Helicobacter* spp. prevents C9orf72-mutant mice from early death and positively influences their underlying systemic inflammation and autoimmunity. There has been no clinical study on the gut microbiome of FTD patients reported.

## Role of nutrition, sedentary lifestyle, sleep deprivation and circadian rhythms on the gut microbiome

Gut microbiome is hypersensitive to external factors associated with an unhealthy lifestyle, such as nutrition, exercise, sleep deprivation, sedentary behavior, and circadian rhythm disorders, all of which are essential elements in controlling healthy aging and prolonging life expectancy (Askarova et al., 2020; Du et al., 2021). Western dietary habits are high in fat and carbohydrate, which can impact behavior and cause shifts in the microbiome composition of high-energy diet mice, with higher *Clostridiales* and lower *Bacteroidales*, which are associated with poor cognitive adaptability (Magnusson et al., 2015). Preclinical research demonstrated that a high-fat diet (HFD) in mice alters the intestinal microbiome composition, with Firmicutes being increased and Bacteroidetes being reduced, implying that it has an impact on the progression of obesity in response to a HFD (Jo et al., 2021). Another study found that HFD alters gut microbiome composition in 3xtg mice, with elevated levels of Firmicutes-to-Bacteroidetes and a decrease in Bifidobacteriaceae, as well as the presence of several bacterial species such as Anaeroplasmataceae, Christensenellaceae, Ruminococcaceae, and Turicibacteraceae. This study also found that HFD contributed to cognitive deficits by causing cell damage and declining neuron cell death via the deactivation of the Nrf2 signaling pathway (Sanguinetti et al., 2018).

A sedentary lifestyle has been associated with severe diseases, including cancer, coronary artery disease and diabetes (Bressa et al., 2017). Sedentary behavior has been associated with poor glycemic regulation in brain function and an increased risk of death. The author propose that treating sedentary behavior with intermittent moderate-intensity exercise could help to prevent cognitive impairment by lowering glycemic variations (Wheeler et al., 2017). Sedentary lifestyle also has an effect on the gut microbiome, with a decrease in butyrate-producing bacteria such as *Butyrivibrio proteoclasticus* and *Marvinbryantia formatexigens*, and an increase in pro-inflammatory microbes, including *Clostridium*, *Eubacterium*, and *Roseburia*, which is positively correlated with an increase of Aβ plaques in the hippocampus and causes the etiology of AD in APP/PS1 transgenic mice (Abraham et al., 2019). Furthermore, clinical research discovered that sedentary women have lower proportions of health-promoting microbes, such as *Akkermansia muciniphila, Faecalibacterium prausnitzii*, and *Roseburia hominis*, than active women (Bressa et al., 2017).

Concurrently, global data show that sleep deprivation enhances the risk of age-related diseases, and recent studies suggest the GM may contribute to this phenomenon (Anderson et al., 2017). Preclinical research has shown that sleep deprivation increases body fat and causes specific alterations in the intestinal microbiome, with Lachnospiraceae and Ruminococcaceae increasing and Lactobacillaceae decreasing, which promote intestinal permeability, inflammation in adipose tissue and insulin sensitivity in mice (Poroyko et al., 2016). There is a finding that poor sleep quality is linked to a reduced abundance of *Verrucomicrobia* and *Lentisphaerae* and suggests that there may be a connection between sleep deprivation, gut microbiome, and cognitive accessibility in the healthy elderly (Anderson et al., 2017). Benedict et al. (2016) discovered that short-term sleep deprivation has mild effects on the gut microbiome, with Firmicutes: Bacteroidetes ratio and Coriobacteriaceae and Erysipelotrichaceae being upregulated, while Tenericutes are downregulated after two days of partial sleep deprivation.

Circadian rhythm regulation is critical in healthy people who are influenced by cosmic events such as light-dark cycles and sleep-wake cycles as well as lifestyles (Farhud and Aryan, 2018). These changes result in circadian rhythm disorders (CRD), which increase the prevalence of mental illnesses such as depression and physiological issues (Farhud and Aryan, 2018). Disruptions of normal circadian rhythms and sleep cycles are consequences of aging and have long been thought to be symptoms of many neurodegenerative conditions (Musiek and Holtzman, 2016). There is evidence that circadian rhythms influence the intestinal microbiome through microbial byproducts including amines, butyrate, polyphenolic compounds, and vitamins (Parkar et al., 2019). In a preclinical study, researchers discovered that disrupting the CRD alters the gut microbiome composition in mice, with *Ruminococcus torques* being increased, a microbe that plays a role in lowering gut barrier integrity, and *Lactobacillus johnsonii* being reduced, a bacterium that aids in maintenance of the intestinal epithelial cell layer (Deaver et al., 2018). Therefore, adequate sleep and a nutritious diet appear to be critical for maintaining gut microbiome balance.

## Targeting gut microbiota as an intervention to delay aging and neurodegenerative diseases

Comprehensive knowledge about the role of the gut microbiome in aging and the emergence of neurological disorders create the potential for new novel interventions for achieving healthy aging (Mancuso and Santangelo, 2018). Gut microbiome interventions have led to growing demand, and the research in this field is constantly evolving. Dietary and probiotic administration have been researched as potential therapeutic approaches for age-associated diseases through changes in gut microbiome composition, with promising findings. Recently, activity has been shown to reshape GM, another factor contributing to the potential benefits of this intervention approach to age-associated diseases (Porras et al., 2018). Here, we summarize the interventions of gut microbiome modulation in both preclinical studies in an animal model (Table 2) and clinical studies in humans (Table 3).

**TABLE 2:** Modulation of the gut microbiome by different types of interventions in preclinical studies.

Interventions	Models	Methodological approach	Main findings	References
Probiotic administration	senescence-accelerated mouse prone 8 (SAMP8 mice)	ProBiotic-4 (*Bifidobacterium* and *Lactobacillus*) (12-weeks)	↑ Firmicutes/Bacteroidetes ↓ Proteobacteria, *Pseudomonas* ↑ Cognitive dysfunction, memory deficits, glial activation, and neuronal injury ↓ Interleukin-6 (IL-6) and tumor necrosis factor-α (TNF-α) ↓ Lipopolysaccharide (LPS), toll-like receptor 4 (TLR4) and nuclear factor-kB (NF-kB)	Yang et al., 2020
	>78 weeks (older C57BL/6J male mice)	Human-origin probiotic cocktail (five *Lactobacillus* and five *Enterococcus*) (10 weeks)	↑ Firmicutes ↑ *Rumminoccocaceae* ↑ *Clostridiales* ↓ *Verrucomicrobiaceae* ↓ *Erysipelotrichaceae* ↓ Inflammation, leaky gut, metabolic disorders, gut dysbiosis, and physical deterioration	Ahmadi et al., 2020
	Aβ (1–42) injected rats.	Probiotics (*Bifidobacterium lactis*, *Bifidobacterium longum, Lactobacillus acidophilus*, and *Lactobacillus fermentum*) (8 weeks)	↑ *Lactobacillus* ↑ *Bifidobacterium* ↑ Learning and spatial memory deficits ↓ Malondialdehyde (MDA) and superoxide dismutase (SOD) (oxidative stress biomarkers)	Azm et al., 2018
	3xTg-AD mice	SLAB51 (*Streptococcus thermophilus*, bifidobacterial and lactobacilli) (4 months)	↑ *Bifidobacterium* spp. ↓ *Campylobacterales* ↓ Brain damage and Aβ aggregates ↑ Cognitive function	Bonfili et al., 2017
Fecal microbiota transplantation (FMT)	(ADLP^APT^) transgenic mouse model of AD	ADLP^APT^ mice administrated with fresh feces of WT mice (16 weeks)	↑ Bacterial diversity ↑ Aβ plaques and neurofibrillary tangles ↓ Gut barrier integrity, chronic systemic inflammation	Kim et al., 2020
	APPswe/PS1dE9 transgenic (Tg) mice and wild-type (WT) mice	Tg + FMT administrated with stool from WT mouse pellets	↑ *Bacteroidetes* ↓ *Proteobacteria* and *Verrucomicrobia* ↑ Cognitive deficits, synaptic plasticity ↓ Aβ40 and Aβ42 levels, tau protein phosphorylation ↓ COX-2 and CD11b levels	Sun et al., 2019b
Dietary intervention	Asymptomatic APOE4 transgenic (E4FAD) mice	Prebiotic diet containing inulin vs. control diet containing cellulose (16 weeks)	↑ *Prevotella* and *Lactobacillus* ↓ *Escherichia*, *Turicibacter*, and *Akkermansia* ↑ SCFAs levels, tryptophan-derived metabolites ↓ Inflammatory gene expression	Hoffman et al., 2019
	(APP/PS1) transgenic (Tg) mice and wild-type (WT) mice	Fructooligosaccharides (FOS) vs. cellulose (CMC-Na) (6 weeks)	↑ *Actinobacteria, Lactobacillus* ↓ *Proteobacteria, Epsilonproteobacteria, Helicobacteraceae*, and *Deferribacteraceae* ↑ Cognitive impairments ↑ Expression synapsin I and postsynaptic density protein 95 (PSD-5) levels	Sun et al., 2019a
	C57BL/6 ApoE knockout mice (ApoE-/-) and wild-type mice	Sesamol (0.05%, w/v, in drinking water) vs. high-fat diet (10 weeks)	↑ *Bacillales*, *Fusobacterium*, and *Lactococcus* ↑ SCFAs production ↑ Cognitive deficits, synapse ultrastructure ↓ Aβ aggregation, gut barrier damages and systemic inflammation	Yuan et al., 2019
	APP/PS1 double-transgenic mice (APP/PS1) and wild-type (WT) mice	Jatrorrhizine (JAT) at high and low dose vs. saline vs. donepezil hydrochloride monohydrate (DONE) (24 weeks)	↑ Firmicutes ↑ Bacteroidetes ↓ Aβ plaques in the cortex and hippocampus ↑ Learning and memory deficits	Wang N. et al., 2019
	Tg2576 mouse model of AD and wild-type (WT)	Calorie restriction (VR) vs. *ad libitum* (AL) (12 months)	↑ *Clostridium sensu stricto 1* ↑ *Lachnospiraceae NK4B4* group ↓*Eubacterium xylanophilum* ↓ Aβ deposition in the brain	Cox et al., 2019
Exercise and probiotic	APP/PS1 transgenic mice (*APP*/*PS1*^TG^)	Interval treadmill running (2 weeks) and FRAMELIM (*Bifidobacterium* and *Lactobacillus*, vitamins A and D, omega-3 fatty acids, B1, B3, B6, B9, B12) (20 weeks)	↑ *Lactobacillus johnsonii* ↑ *Bacteroides thetaiotaomicron* ↓ Beta-amyloid plaques in the hippocampus	Abraham et al., 2019
Diet and exercise	C57BL/6NTac mice	Normal vs. HFD and exercise vs. sedentary groups (12- weeks)	↑ *Clostridium* spp. ↑ *Allobaculum* spp. ↑ *Faecalibacterium prausnitzi* ↓ Intestinal inflammatory response	Campbell et al., 2016

Changes (↑: increase; ↓: decrease) in the relative abundance of selected microbial taxa and other main findings in the study.

**TABLE 3:** Modulation of the gut microbiome by different types of interventions in clinical studies.

Interventions	Subjects	Methodological approach	Main findings	References
Probiotic administration	≥65 years old (63 healthy elders)	Randomized double-blind, multicenter clinical trial. *Bifidobacterium bifidum* BGN4 and *Bifidobacterium longum* BORI vs. placebo (12 weeks)	↓ *Eubacterium* ↓ Clostridiales ↑ Serum BDNF level ↑ Mental flexibility and alleviate stress	Kim et al., 2021
	60–93 years old (20 Alzheimer’s disease patients)	Multispecies probiotics (*Lactobacillus*, *Lactococcus, Bifidobacterium*) (4 weeks)	↑ *Faecalibacterium prausnitzii* ↑ Serum kynurenine concentrations	Leblhuber et al., 2018
Dietary intervention	65–79 years old (612 older adults)	A randomized single-blind, multicenter controlled trial. Mediterranean diet (MD) vs. control group (1-year)	↑ *Eubacterium* ↑ *Bacteroides thetaiotaomicron* ↓ *Ruminococcus torques* ↓ Frailty ↑ Cognitive function ↑ Short or branch chained fatty acid production ↓ Secondary bile acids, carbon dioxide, ethanol and p-cresols production	Ghosh et al., 2020
Physical activity	>61 years old (897 overweight elderly)	Comparative study. Daily/regular exercise vs. never/rare exercise group	↑ *Turicibacteraceae* ↓ *Pseudomonadaceae* ↑α-diversity of gut microbiota	Zhu et al., 2020
	65–92 years old (338 community-living Japanese)	Cross-sectional and observational design. Physical activity was measured using a uniaxial acceleration sensor (1 month)	↑ *Bacillaceae* ↓ *Fusobacteriaceae* ↑ Bowel function ↑ Mechanical stimulation of intestinal movements	Aoyagi et al., 2019
	>65 years old (32 sedentary women)	Non-randomized comparative trial. Aerobic exercise training vs. trunk muscle training (12 weeks)	↑ *Bacteroides* ↑ Cardiorespiratory fitness	Morita et al., 2019
	62–76 years old (33 Japanese men)	Randomized crossover trial. Endurance exercise (5 weeks)	↓ *Clostridium difficile* ↑ *Oscillospira* (related to cardiometabolic risk factors)	Taniguchi et al., 2018

Changes (↑: increase;↓: decrease) in the relative abundance of selected microbial taxa and other main findings in the study.

Several studies have discovered that probiotics improve gut epithelium integrity, prevent barrier degradation, reduce pro-inflammatory responses, and prevent the initiation or proliferation of neuroinflammation and neurodegeneration (Frasca and Blomberg, 2015; Plaza-Díaz et al., 2017). *Bifidobacterium* and *Lactobacillus* species are frequently found in probiotic formulations, extensively designed to promote human health and classified as Generally Regarded as Safe (GRAS) (Fijan, 2014). Numerous preclinical studies have also highlighted the probiotic potency of lactobacilli and bifidobacteria (Table 2; Bonfili et al., 2017; Kobayashi et al., 2017; Azm et al., 2018; Tan et al., 2020; Yang et al., 2020). Probiotic administration demonstrated to significantly alter gut microbiome composition, decrease interleukin-6 (IL-6) and tumor necrosis factor-α (TNF-α), lower LPS, decreased toll-like receptor 4 (TLR4) and nuclear factor-kB (NF-kB) expression, and improve cognitive function (Savignac et al., 2015; Azm et al., 2018; Leblhuber et al., 2018; Yang et al., 2020). These findings imply that probiotics could be a potential intervention for neurodegenerative diseases. Another intervention aimed at the gut microbiome is fecal microbiota transplantation (FMT), which involves transferring fecal from a healthy donor into the GIT of a patient to improve gut microbiome diversity and roles in patients with gut dysbiosis (Gulati et al., 2020), including *Clostridioides difficile* infection (CDI) (Nood et al., 2013) and inflammatory bowel disease (IBD) (Sokol et al., 2020). Preclinical studies in animal models revealed that FMT not only restores the microbial population but also improves synaptic plasticity and cognitive deficits, particularly spatial learning and memory, reduces the amyloid-β (Aβ) deposition and tau protein phosphorylation and modifies abnormalities activity in gut macrophages and circulating inflammatory monocytes in the blood (Sun et al., 2019b; D’Amato et al., 2020; Kim et al., 2020). Application of FMT to a patient with AD and PD has not yet been reported (Evrensel and Ceylan, 2016). FMT is a safe therapeutic approach with minor complications due to living microorganisms and related metabolites (Bonfili et al., 2021). In contrast, FMT has a few limitations, such as donor microbe variations, the adverse effects of pathobiont transmission, and unidentified long-term effectiveness. Interestingly, an alternative strategy has been proposed: to use donors rich in butyrate-producing microbes to improve gut microbiome diversity (Wilson et al., 2019).

However, dietary intervention is one of the most effective interventions for altering the gut microbiome due to its safety and is more beneficial than drug-based therapies. Diets high in carbohydrates, saturated fat, and processed foods may increase health risks by reducing microbiome diversity, intestinal barrier function, promote neuroinflammation, and cognitive decline (Bonfili et al., 2021). Calorie restriction (CR) has been discovered as one of the most effective non-genetic nutritional modifications for extending longevity and preventing age-related diseases in many species (Kapahi et al., 2017; Mattison et al., 2017). Several other animal studies support diet-based therapeutic interventions such as calorie restriction, probiotic-enriched foods, and consumption of digestion-resistant fibers (Cox et al., 2019; Hoffman et al., 2019; Sun et al., 2019a; Wang S. et al., 2019; Yuan et al., 2019). The Mediterranean diet (MD) is a healthy diet that emphasizes vegetables, legumes, nuts, fruits, unsaturated fatty acids, and polyphenols (Zhang et al., 2020). In addition, MD has been linked to a longer lifespan and a lower risk of fragility (Ghosh et al., 2020), cardiovascular diseases (Estruch et al., 2018), and cognitive deterioration (Valls-pedret et al., 2015) and cancer in the elderly (Toledo et al., 2015).

Physical activity has also been proven to extend life expectancy and reduce the detrimental of age-associated disorders (Cabanas-Sánchez et al., 2018; Chudasama et al., 2019). Despite these significant benefits, physical exercise declines with age, and most older people are sedentary. Maintaining physical exercise has been demonstrated to alter gut microbiome composition and enhance the numbers of butyrate-producing microbes (Allen et al., 2018), improve gut barrier integrity, and attenuate gut inflammation in animal models, thereby improving their health status (Table 2; Campbell et al., 2016). Previously, high-fitness adults living in a community were found to have higher frequencies of Bifidobacteriales and Clostridiales species than poor-fitness individuals (Castro-Mejía et al., 2020). Physical exercise has been shown in clinical studies to significantly affect the microbiome’s function and composition, resulting in more extended longevity in people with multimorbidity (Allen et al., 2018; Cabanas-Sánchez et al., 2018; Chudasama et al., 2019).

## Conclusion and future perspective

New novel approaches to healthy aging are required as the population ages, life expectancies increase, and the burden of age-related health issues grows. The gut microbiome is responsible for a variety of both pathological and physiological mechanisms, and its role in aging and neurological processes has been underlined as a potential target for anti-aging interventions. Numerous reviews have been published on the correlation between the gut microbiome and aging and therapeutic interventions. Recent preclinical studies have revealed the efficacy of microbiota-based intervention approaches, but more advanced human studies are required to support the hypothesis. Therefore, more extensive sample size studies are needed, including demographic, lifestyle, and biological factors that may influence microbial composition in humans and advanced high-throughput sequencing analysis. Understanding the interaction between aging, gut microbiome, neurodegenerative diseases, and interventions targeting the gut microbiome is critical in this context, as this knowledge could lead to a discovery that expands the possibilities for delaying aging and combating neurodegenerative diseases.

## Author contributions

SM developed the concept and revised the manuscript. HH analyzed the literature, wrote and edited the draft of the manuscript, drew the diagrams, and prepared the tables. Both authors contributed to the article and approved the submitted version.
